# Natural Variation in *OsMKK3* Contributes to Grain Size and Chalkiness in Rice

**DOI:** 10.3389/fpls.2021.784037

**Published:** 2021-11-25

**Authors:** Yinghua Pan, Lei Chen, Yan Zhao, Haifeng Guo, Jingcheng Li, Muhammad Abdul Rehman Rashid, Chunju Lu, Weiyong Zhou, Xingka Yang, Yuntao Liang, Hao Wu, Dongjing Qing, Lijun Gao, Gaoxing Dai, Danting Li, Guofu Deng

**Affiliations:** ^1^Guangxi Key Laboratory of Rice Genetics and Breeding, Rice Research Institute, Guangxi Academy of Agricultural Sciences, Nanning, China; ^2^State Key Laboratory of Crop Biology, Shandong Key Laboratory of Crop Biology, College of Agronomy, Shandong Agricultural University, Tai’an, China; ^3^Beijing Key Laboratory of Crop Genetic Improvement, State Key Laboratory of Agrobiotechnology, College of Agronomy and Biotechnology, China Agricultural University, Beijing, China; ^4^Department of Bioinformatics & Biotechnology, Government College University Faisalabad, Faisalabad, Pakistan; ^5^Guangxi Lvhai Seed Co., Ltd., Nanning, China; ^6^Guangxi Crop Genetic Improvement and Biotechnology Laboratory, Guangxi Academy of Agricultural Sciences, Nanning, China

**Keywords:** *OsMKK3*, grain size, rice domestication, haplotype, artificial selection, genetic interaction

## Abstract

Rice (*Oryza sativa* L.) is an important staple food crop for more than half of the world’s population. Enhancing the grain quality and yield of rice to meet growing demand remains a major challenge. Here, we show that *OsMKK3* encode a MAP kinase kinase that controls grain size and chalkiness by affecting cell proliferation in spikelet hulls. We showed that *OsSPL16*, *GS5*, and *GIF1* have a substantial effect on the *OsMKK3*-regulated grain size pathway. *OsMKK3* has experienced strong directional selection in *indica* and *japonica*. Wild rice accessions contained four *OsMKK3* haplotypes, suggesting that the *OsMKK3* haplotypes present in cultivated rice likely originated from different wild rice accessions during rice domestication. *OsMKK3*-Hap1, *gs3*, and *gw8* were polymerized to enhance the grain length. Polymerization of beneficial alleles, such as *OsMKK3*-Hap1, *gs3*, *gw8*, *fgr*, *alk*, *chalk5*, and *wx*, also improved the quality of hybrid rice. Overall, the results indicated that beneficial *OsMKK3* alleles could be used for genomic-assisted breeding for rice cultivar improvement and be polymerized with other beneficial alleles.

## Introduction

Rice (*Oryza sativa* L.) is an important staple food crop for over half of the world’s population. Grain yield is determined by three key factors: grain weight, number of grains per panicle, and number of panicles per plant. Grain size is an important breeding target that affects both yield and appearance quality and is determined by the length, width, and thickness of the grain. Studies of grain size can provide new insights that could be used to improve the yield and quality of rice and the rice domestication process (Shomura et al., 2008).

Many quantitative trait loci (QTLs) of grain length have been mapped, and some of these QTLs, such as *GS3*, *OSLG3*, *qGL3, SMG1*, and *OsSPL13*, have been cloned as well. *GS3* encodes a protein of 232 amino acids with a putative PEBP-like domain, a transmembrane region, a putative TNFR/NGFR family cysteine-rich domain, and a VWFC module that is a negative regulator of grain size (Fan et al., 2006). *GS3* is an evolutionarily important gene controlling grain size in rice by a C to A mutation in the second exon of *GS3* (A allele) (Takano-Kai et al., 2009). *GL3.1* encodes a protein phosphatase kelch (PPKL) family — Ser/Thr phosphatase that affects the phosphorylation of proteins in the spikelet and accelerates cell division. *GL3.1* increases the grain length and results in higher yields (Qi et al., 2012). *OSLG3* was found to encode an AP2 domain class transcription factor that positively regulates grain length and improves rice yield without affecting grain quality. *OSLG3* alleles in *indica* and *japonica* evolved independently from distinct ancestors (Yu et al., 2017). *OsSPL13* encodes a plant-specific transcription factor that regulates the cell size of the grain hull and enhances rice grain length and yield (Si et al., 2016). *SMG1* is a mitogen−activated protein kinase kinase 4 that regulates grain size through its effect on the MAPK pathways and brassinosteroids (Duan et al., 2014). A study examining the function of *OrMKK3* in *Oryza officinalis* Wall. ex Watt revealed that the plant height was lower, the grains were shorter, and the number of tillers was higher in plants overexpressing *OrMKK3* than wild-type plants of the Nipponbare (Nip) cultivar (Pan et al., 2021). There is thus a need to study the genetic and molecular bases of grain size (Mao et al., 2010).

Rice breeders use natural variations in grain size to improve yield and quality, but only a few alleles of genes regulating grain shape have been widely used. Class A haplotypes of *OsLG3* show a longer grain phenotype compared with class B haplotypes of *OsLG3* in several cultivars (Yu et al., 2017). Hap-SLG of *OsLG3b* shows a longer grain phenotype than Hap-NIP and has much breeding potential for increasing grain length in *indica* (Yu et al., 2018). Application of the *qgl3* allele can increase the grain yield through its positive effect on grain length, filling, and weight (Zhang et al., 2012). Xia et al. (2018) pyramided the *GW7* allele from TFA and *gs3* to develop new high-yielding *indica* hybrid rice varieties. Liu et al. (2018) combined the *OsMADS1lgy3* allele with high yield-associated *dep1-1* and *gs3* alleles, which enhanced both rice yield and quality. More characterizations of gene–coding sequence–haplotype (gcHap) diversity would facilitate basic research and improvement of rice (Zhang et al., 2021).

Here, *OsMKK3* was shown to be a positive regulator of grain length and height in rice. Characterization of natural variations in the *OsMKK3* coding sequence (CDS) associated with grain length and chalkiness and favorable alleles could provide useful genetic resources for improving rice cultivars. By pyramiding *OsMKK3*, *fgr*, *wx*, *alk*, and *gs3* alleles, Zhou et al. (2019) developed the new high-yielding *indica* hybrid rice variety Wantaiyou3158, which had higher yield and grain quality.

## Materials and Methods

### Plant Materials and Measurement of Grain and Yield Traits

Yexiang maintainer line (YXB) is an excellent quality hybrid rice parent that has been used to produce more than 30 varieties. The varieties have been planted widely in southern China. Rice was planted under natural field conditions at the Rice Research Institute of Guangxi Academy of Agricultural Sciences, Nanning, China in the summers of 2015–2021 (22.85°N, 108.26°E). The distance between plants within rows was 16.7 cm, and the distance between plants in separate rows was 20 cm. Field management, including irrigation, fertilizer application, and pest control, followed normal agricultural practices. Fully filled grains were used for measurements of grain width, length, and weight with a Wanshen SC-G automatic seed test system. All trait measurements were repeated at least 3 times. A total of 342 Guangxi common wild rice core germplasm accessions (Pan et al., 2018), a total of 419 Guangxi core germplasm landrace accessions (Yang et al., 2018), and 94 improved varieties (Supplementary Table 1) were used in this study.

### Vector Construction and Rice Transformation

To generate the overexpression vector, the open reading frame of *OsMKK3* was amplified from the cDNA of Nip (Supplementary Table 2) and cloned into the pMDC32 vector. sgRNA-Cas9 plant expression vectors were constructed as described previously (Mao et al., 2013).

### Gene Genotyping by PARMS

The PARMS is a KASP-like SNP genotyping technique combined with ARMS, which is also referred to as allele-specific PCR (Newton et al., 1989; Heim and Meyer, 1990). The primer sequence information of SNP markers for rice was obtained from the rice 3K project (RFGB^1^). The primers were both designed by Primer Premier 5.0 (Supplementary Table 3). Genotyping tests were carried out with PARMS (Gentides, China). The PCR reactions were conducted in 384-well PCR plates for PARMS genotyping. The 5-μl PCR reaction system contained 2 × PARMS PCR reaction mix, each allele-specific primer (150 nM), locus-specific primer (400 nM), and 1.4 μl of alkaline lysis DNA template. 5-μl of mineral oil was added into each well of the PCR plate to prevent evaporation of the PCR mix. The thermal cycler program of PARMS was denaturation at 95°C for 15 min and 10 cycles of 95°C for 20 s, followed by annealing at 65°C for 1 min. The temperature was then decreased 0.8°C per cycle to the annealing temperature at 57°C, which was followed by 32 cycles of denaturation at 95°C for 20 s and annealing at 57°C for 1 min. The well plate was read using a TECAN Infinite M1000 plate reader; SNP calling and plots were conducted using an online software SNP Decoder^2^ combined with manual modification. Sequence analysis was performed following the method provided by Wuhan Gentides Biotech Co., Ltd.

### DNA Extraction, RNA Extraction, Expression Analysis, and RNA-Seq

An EasyPure^®^ Plant Genomic DNA Kit (Trans, China) was used for genomic DNA extraction. Panicles of YXB and YXB-Cr line of different lengths (3, 5, 10, 15, 20, and 25 cm) at the booting stage were flash-frozen in liquid nitrogen. Total RNA from the aforementioned tissues was extracted using a Takara MiniBEST Plant RNA Extraction Kit per the manufacturer’s instructions (Takara, Catalog no. 9769). Total RNA (2.0 μg) was used for cDNA synthesis with a PrimeScript^TM^ RT Reagent Kit with gDNA Eraser (TransScript^®^ II One-Step RT-PCR SuperMix, Tran, Catalog no. AH411-02). The resulting cDNA samples were diluted five times and used as templates for PCR. We performed qRT-PCR using a TransStart^®^ Green qPCR SuperMix and a CFX96 RealTime system (Bio-Rad, Hercules, CA, United States) following the manufacturer’s instructions. qRT-PCR was performed using 10-μL mixtures containing 5 μL of 2 × Green qPCR MasterMix, 1 μL of cDNA, 0.25 μL of each primer (10 μM), and 3.5 μL of ddH_2_O. Amplification steps were 95°C for 30 s, 40 cycles of 95°C for 5 s, and 60°C for 30 s, followed by 65°C for 5 s, 95°C for 15 s, 60°C for 30 s, and 95°C for 15 s. Each experiment was repeated at least three times. The qRT-PCR analysis was performed using the ΔΔ*C*t method. Details on gene-specific primers used for real-time PCR are provided in Supplementary Table 1. qRT-PCR was performed in triplicate for each sample, and the *Ubiquitin* gene (*LOC_Os03g13170*) (Chen et al., 2021) was used as a control (^∗^*P* < 0.05, ^∗∗^*P* < 0.01; Student’s *t-*test).

RNA samples used for RNA-seq analysis were prepared from 10 and 20-cm panicles of YXB and YXB-cr line grown under normal field conditions with three biological replicates. RNA library sequencing was performed on an Illumina Hiseq^TM^ 2500/4000 platform by Gene *Denovo* Biotechnology Co., Ltd. (Shanghai, China). Sequence analysis was performed using the method provided by Majorbio. Additional detailed information is provided on the Majorbio website^3^.

### Selection of Germplasm and Phylogenetic Analysis

The phylogenetic tree of rice core germplasm landrace accessions, *Oryza rufipogon* wild rice core germplasm accessions, and improved varieties was constructed based on SNPs from the rice 3K project (RFGB, see Text Footnote 1). A total of 419 rice core germplasm landrace accessions were from Guangxi province (Yang et al., 2018). A total of 351 *O*. *rufipogon* wild rice core germplasm accessions were from Guangxi province (Pan et al., 2018). A total of 96 improved varieties were conserved in the Rice Research Institute, Guangxi Academy of Agricultural Sciences. A neighbor-joining variety tree of rice varieties was constructed using MEGA 7.0. The number of bootstrap replicates was 1,000 (Kumar et al., 2016). The phylogenetic tree was visualized with the iTOL online tool (Letunic and Bork, 2019). An independent samples *t*-test was performed to identify differences in phenotypes between groups and control.

### Nucleotide Diversity Analysis

The genomic sequences of 2,644 cultivated and 42 wild accessions were available from the 3K Rice Genome Project (3KRGP) (Alexandrov et al., 2015; Wang et al., 2018) and OryzaGenome^4^, respectively. The average nucleotide diversity (π and θ) and Tajima’s *D* for each subpopulation in *OsMKK3* and flanking regions (40-kb) were calculated using DnaSP 5.10 (Librado and Rozas, 2009). The nucleotide diversity curves were acquired using a 40-bp window and 10-bp step length. Population differentiation statistics (*F*_*ST*_) were calculated using VCFtools software (Danecek et al., 2011) with a 3,000-bp window and 300-bp step length.

### Histological Analysis

To observe the morphology of starch granules in the grain of Nip, the transgenic line, YXB, and YXB-Cr line. Milled rice grains were transversely cut in the middle with a sharp knife. Samples were then cleaned, placed in an electron microscope fixator at room temperature for 2 h, and then transferred to 4°C for preservation. The samples were fixed, dehydrated, and dried with a critical point drier. Samples were attached to conductive carbon film double-sided tape and coated with gold under vacuum for 30 s. Milled rice grains for scanning electron microscopy were transversely cut in the middle with a sharp knife, attached to conductive carbon film double-sided tape, and coated with gold under vacuum for 30 s. The morphology of starch granules in the belly part of the endosperm was examined with a scanning electron microscope (Hitachi, SU8100, Wuhan Servicebio Technology) at an accelerating voltage of 12 kV. The analysis was based on at least three biological replications of mounted specimens. All procedures were carried out per the manufacturer’s protocol.

## Results

### *OsMKK3* Regulates Grain Size, the Accumulation of Starch, and the Expression of Other Genes Involved in the Production of Rice

**FIGURE 1:**
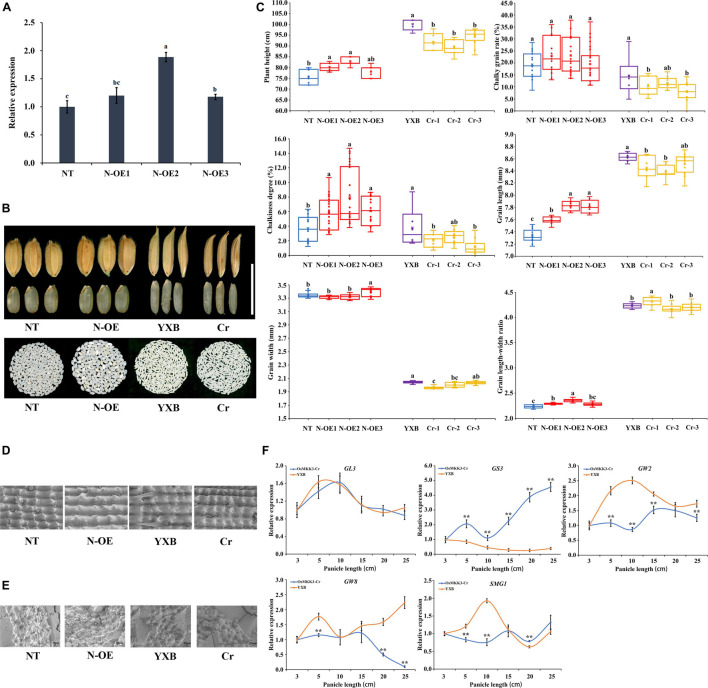
Identification of *OsMKK3.* **(A)** Relative expression of *OsMKK3* in NT and transgenic plants. **(B)** Functional validation of the role of *OsMKK3* in grain size and chalkiness by genetic transformation. **(C)** Comparison of the grain length, grain width, chalkiness rate, chalkiness score, and plant height between transgenic plants and NT. Data represent mean ± S.E.M. (*n* = 12). Student’s *t*-tests were used to generate *P*-values. **(D)** The epidermal cells of rice husk in transgenic plants and NT. Bar = 1 cm. **(E)** Scanning electron microscope images of chalkiness in transgenic plants and NT. Bar = 50 μm. **(F)** Relative expression of important genes in NT and transgenic plants. a, b, and c represent significant difference at 5% probability level. ** represents significant difference at 1% probability level.

According to our previous study (Pan et al., 2021), *OrMKK3* of *O. officinalis* Wall. ex Watt affects the morphology and grain size of rice. We performed a series of studies of *OsMKK3* in rice to determine its function. Overexpression constructs containing the *OsMKK3* CDS from *Nip* (N-OE) driven by the 35S promoter from tobacco cauliflower mosaic virus (CaMV35S) were separately introduced into *Nip*. N-OE lines showed significantly longer grains, higher grain length-width ratio, chalkiness degree, and greater higher plant height (Figures 1A–C). N-OE lines had a higher chalky grain rate than the no transgene line (NT). And N-OE lines showed few changes in grain width. In general, the expression of *OsMKK3* regulated grain size, chalkiness, and height. In addition, we used a CRISPR-Cas9 system for targeted gene mutation of *OsMKK3* in the Yexiang maintainer line (YXB) (Figure 1A). The target sequence (5′-AAATCTCAAGGGTGAGGCAAA-3′) was at sites +666–+667 within the fourth exon encoding the C-terminal of *OsMKK3* (amino acid residues 222). These deletions lead to frameshifting mutations that result in differences in the C-terminal of *OsMKK3* and incomplete peptides of *OsMKK3*; these changes result in a loss of *OsMKK3* function. The grain of transgenic plants (Cr) was smaller than the wild-type YXB (Figures 1B,C). The Cr line showed a significant reduction in grain length, grain width, grain length-to-width ratio, and chalky rice rate (Figures 1B,C). This finding was consistent with the reduced production of rice and the change in chalkiness caused by the loss of *OsMKK3* function. The results of the histological analysis indicated that the chaff of N-OE increased in size compared with that of NT, and the chaff of Cr became shorter compared with that of NT and YXB. Scanning electron microscopy images of transverse sections of N-OE grains indicated that this endosperm was filled with loosely packed, small, and spherical starch granules with large air spaces (Figure 1D), and the Cr endosperm consisted of densely packed, large, and irregularly shaped polyhedral starch granules (Figure 1E). These results suggest that *OsMKK3* regulated the accumulation of starch and affected grain size. Furthermore, real-time quantitative PCR (qRT-PCR) analysis of *GL3*, *GW2*, *GW8*, *SMG1*, and *GS3* was performed in the Cr line and YXB to study the development of spikes. These genes regulate grain size and cell cycle time (Figure 1F). The expression patterns of *GL3* and *PPKL1* were the same in YXB and the Cr line at 3, 5, 10, 15, 20, and 25 cm panicle length. The expression of *GS3* was lower in YXB than in the Cr line. The expression of *OsLG3* and *SMG1* was higher in YXB than in the Cr line. These results indicate that the knockout of *OsMKK3* affected *GS3, OsLG3*, and *SMG1* expression. In short, *OsMKK3* played an important role in cell development in rice, regulated genes involved in grain size and cell cycle time, and affected the grain size and accumulation of starch.

### *OsMKK3* Regulates Genes in the Early Spike Development Stage to Control Grain Size and Chalkiness

**FIGURE 2:**
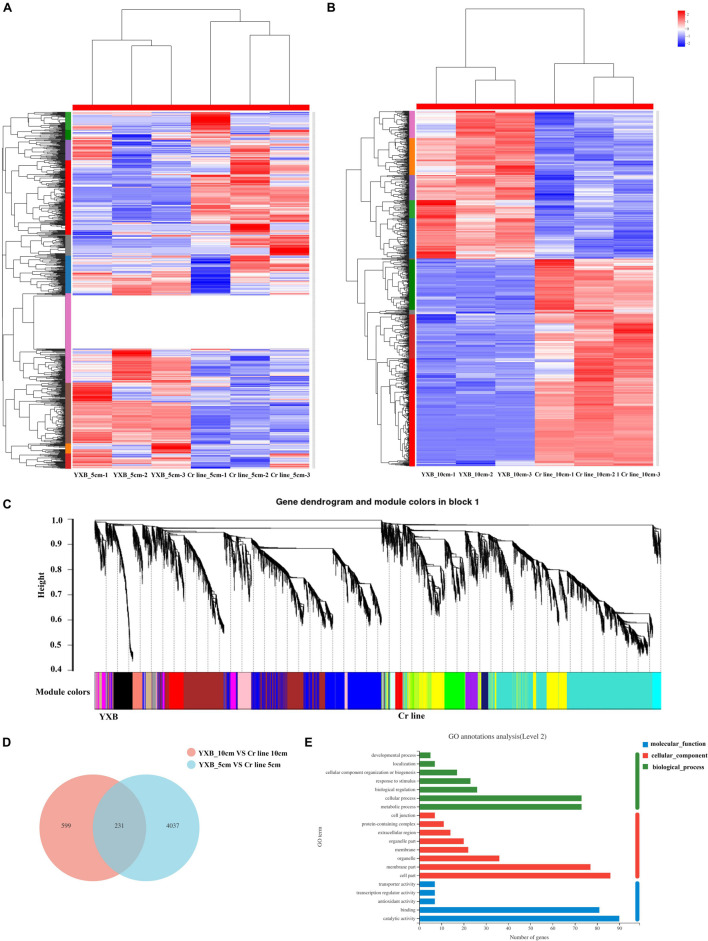
*OsMKK3* regulates genes in the early spike development stage. **(A)** Hierarchical clustering of the DEGs for the YXB and the Cr line in the spike development stage. **(B)** Weighted gene co-expression network analysis of DEGs identified from YXB and the Cr line in the spike development stage. **(C)** Common DEGs expressed in YXB_5cm_vs_Cr line_5cm and YXB_10cm_vs_Cr line_10cm. **(D)** GO enrichment analysis of common DEGs. **(E)** GO term of common DEGs.

To analyze the effects of *OsMKK3* on the transcriptome of rice grain and spike, we studied 5- and 10-cm spikes in YXB and the Cr line. There were three biological replicates per condition for RNA-seq. Overall, more than 83.86 Gb of clean data were generated, and these were used for mapping onto the Os-Nipponbare-Reference-IRGSP-1.0 genome^5^. The results showed that the Q30 base percentage was above 93.03%. A total of 40,225 genes were expressed, 35,193 genes were identified as known genes, and 5,032 genes were identified as new genes. YXB_5cm was used as a control in the group YXB_5cm_vs_Cr line _5cm. The total differential expression analysis revealed 4,268 differentially expressed genes (DEGs), including 1,496 up-regulated DEGs and 2,772 down-regulated DEGs (Figure 2A). YXB_10cm was used as a control in the group YXB_10cm_vs_Cr line_10cm; the total number of DEGs was 830, including 485 up-regulated DEGs and 345 down-regulated DEGs (Figure 2B). DEGs in the 5-cm spike stage revealed that *OsMKK3* affects the expression of several genes in early spike development to regulate traits such as grain size, height, and starch accumulation. To identify the common target genes of the *OsMKK3* regulatory module, we next compared the genome-wide transcriptional profiles in the developing panicles, spikelet hulls, and starch accumulation of YXB and Cr line plants using weighted gene co-expression network analysis. The network was constructed from the filtered probes, and 18 co-expressed modules were identified. The module detection parameters were as follows: minimum module size 30, the module detection sensitivity deepSplit 2, and cut height for merging of modules 0.25 (meaning that modules whose eigengenes are correlated are merged). For example, the MEblack, MEpink, MEmagenta, MEgreen, MEsalmon, MEcyan, MEbrown, and MEred modules were related to up-regulated DEGs in the grain development stages, especially the MEsalmon module, which was strongly related to grain development; the red and brown modules were negatively related to the very early stage of grain development; and the MEtan, MEgrey60, and MEpurple modules were specifically related to down-regulated DEGs in the grain development stages (Figure 2C). In short, multiple modules were related to one or more grain development and starch accumulation stages associated with *OsMKK3*.

To identify the *OsMKK3* pathways involved in spike development, we analyzed 231 common DEGs expressed in both YXB_5cm_vs_ Cr line_5cm and YXB_10cm_vs_cr line_10cm (Figure 2D). The results indicated that 90 DEGs were up-regulated in YXB_5cm_vs_ Cr line_5cm and YXB_10cm_vs_Cr line_10cm; 124 DEGs were down-regulated in YXB_5cm_vs_Cr line_5cm and YXB_10cm_vs_Cr line_10cm; 12 DEGs were down-regulated in YXB_5cm_vs_Cr line_5cm but up-regulated in YXB_10cm_vs_Cr line_10cm; and 5 DEGs were up-regulated in YXB_5cm_vs_Cr line_5cm but down-regulated in YXB_10cm_vs_Cr line_10cm. To investigate the biological functions of these DEGs, Gene Ontology (GO) enrichment analysis was performed with agriGO. DEGs of cellular process and metabolic process were more enriched compared with other GO terms within biological process. DEGs of cell part were more enriched compared with other GO terms within cellular component. DEGs of catalytic activity were most enriched in molecular function (Figure 2E). In the GO enrichment analysis, 137 DEGs were enriched in cellular component, 86 DEGs were enriched in cell part, and 76 DEGs were enriched in membrane part (Figure 2E). The results indicated that 231 DEGs may be regulated by *OsMKK3* pathways. Most DEGs were enriched in cell development. To determine whether *OsMKK3* affected genes controlling grain size and starch accumulation, we examined 22 genes that have been cloned and identified to be involved in grain size and starch accumulation in the spike development stage. In YXB_5cm_vs_cr line_5cm, there were three up-regulated genes: *OsSPL16*, *GS5*, and *GIF1*. *Wx* was up-regulated in YXB_5cm_vs_cr line_5cm. *OsSPL16* was up-regulated in YXB_10cm_vs_Cr line_10cm. *GS5*, *GIF1*, and *Wx* were not detected in YXB_10cm_vs_Cr line_10cm. *OsSPL16*, *GS5*, *GIF1*, and *Wx* affect the *OsMKK3*-regulated grain size and chalkiness pathway; *OsSPL16* in particular may be up-regulated in the *OsMKK3* pathway. Overall, these results indicate that *OsMKK3* is a key functional factor controlling grain size and chalkiness by regulating a series of genes.

### *OsMKK3* Has Undergone a Selective Sweep During the Domestication of Indica and Temperate Japonica

**FIGURE 3:**
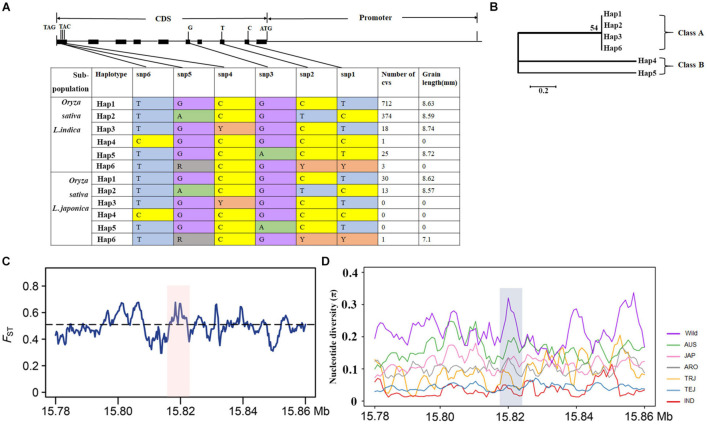
Haplotypes and genomic differentiation of *OsMKK3*. **(A)** Haplotype analysis of *OsMKK3*. Gene structure and natural variation between alleles from NIP. **(B)** Cladogram of six haplotypes. **(C)** Genetic differentiation of *OsMKK3* and major associated loci. Blue and green lines represent the *F*_*ST*_ between *indica* and *japonica*. **(D)** Nucleotide diversity analysis in *OsMKK3* and flanking regions (∼40 kb).

We performed a haplotype analysis of *OsMKK3* in 3,110 cultivated varieties and 446 wild rice samples to investigate natural variation in *OsMKK3* among rice germplasm accessions and identified six main haplotypes (Hap) for *OsMKK3* (Figure 3A). Based on the phylogenetic analysis, the six haplotypes could be classified into two classes: haplotypes 1, 2, 3, and 6 in class A and haplotypes 4 and 5 in class B (Figure 3B). Phenotype analysis showed that cultivars with class A haplotypes had a longer grain phenotype compared with those with class B haplotypes. Hap1 is the main haplotype in both *indica* and *japonica.* We also calculated population differentiation statistics (*F*_*ST*_) for *OsMKK3* and its flanking regions between *indica* and *japonica*. *F*_*ST*_ in *OsMKK3* was above the genome-wide threshold, indicating that there was genetic differentiation in *OsMKK3* between *indica* and *japonica* subspecies (Figure 3C).

To reveal whether selection has acted on *OsMKK3*, 2,644 cultivated and 42 wild accessions were used to analyze the genetic diversity of *OsMKK3* and its flanking regions. Compared with *O. rufipogon*, the nucleotide diversity of *OsMKK3* was significantly lower in *indica* and temperate *japonica*. Tajima’s *D*-values for *indica* and temperate *japonica* were negative and statistically significant (Table 1), suggesting that *OsMKK3* has experienced strong directional selection in these two subpopulations. Tajima’s *D* for cultivated varieties was positive and statistically significant, indicating that *OsMKK3* had high polymorphism and might have experienced balancing selection. Given that directional selection might result in a selective sweep in the flanking region of selected genes, we examined the nucleotide diversity of 40-kb regions flanking *OsMKK3* (Figure 3D). The average nucleotide diversity of *OsMKK3*-flanking regions in *indica* and temperate *japonica* was comparable to that of the *OsMKK3* region but much lower than that in *O. rufipogon* populations. In addition, the Tajima’s *D* of *OsMKK3*-flanking regions in *indica* and temperate *japonica* was also negative and statistically significant. These findings indicated that *OsMKK3* may have undergone a selective sweep during the domestication of *indica* and temperate *japonica* subspecies.

**TABLE 1:** The nucleotide diversity and Tajima’s *D* of *OsMKK3* and its flanking regions.

**Region**	**Parameter**	**AUS (197)**	**ARO (74)**	**IND (1726)**	**Tem-Jap (283)**	**Tro-Jap (364)**	***Japonica* (647)**	**Cultivated (2644)**	**Wild rice (42)**
Upstream 40 kb	S	336	328	265	321	290	296	467	172
	π	0.21429	0.14052	0.04033	0.06541	0.22954	0.16464	0.28399	0.33846
	θ	0.17071	0.20515	0.1187	0.16075	0.15449	0.14187	0.12063	0.2324
	Tajima’s D	0.8159	−1.09583	−1.85917*	−1.84654*	1.48568	0.47486	3.81992**	1.67989
*OsMKK3*	S	55	56	54	53	53	54	76	31
	π	0.18851	0.14442	0.03559	0.06025	0.24019	0.16361	0.28996	0.38024
	θ	0.17071	0.20515	0.11594	0.16075	0.15449	0.14187	0.12131	0.2324
	Tajima’s D	0.31259	−0.97944	−1.79463*	−1.81431*	1.57934	0.42008	3.6679**	1.87876
Downstream 40kb	S	334	321	308	318	280	286	477	174
	π	0.19831	0.13933	0.02858	0.05915	0.25538	0.17581	0.27073	0.36099
	θ	0.17071	0.20515	0.11622	0.16075	0.15449	0.14187	0.12082	0.2324
	Tajima’s D	0.51671	−1.11587	−2.1302**	−1.96754*	1.99581	0.70735	3.50034**	1.73723

* and ** represent significant difference at 5% and 1% probability levels, respectively.

### Selection Leads to Differences in the Main Haplotypes of Wild and Cultivated Rice

**FIGURE 4:**
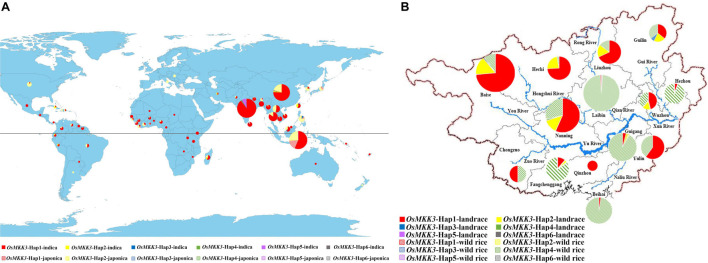
Distribution of the main *OsMKK3* haplotypes in wild rice and cultivated rice in the world and Guangxi province. **(A)** Geographic origin of *OsMKK3.* Hap1–6 is represented by red, yellow, blue, green, purple, and gray, respectively. The *indica* and *japonica* cultivars are denoted by solid and dashed circles, respectively. **(B)** Geographic origin of *OsMKK3* in Guangxi province. Hap1–6 is represented by red, yellow, blue, green, purple, and gray, respectively. The rice landraces and wild rice cultivars are denoted by solid and dashed circles, respectively.

To characterize the geographic distribution of *OsMKK3* haplotypes, we analyzed the geographic distribution of *OsMKK3* in 1,703 cultivated varieties of the 3K project. Most *japonica* accessions were distributed in northern regions, whereas *indica* accessions were mostly distributed in southern regions. Cultivars distributed in China, Sri Lanka, Sierra Leone, Philippines, Malaysia, Madagascar, Korea, Indonesia, and India have more than four *OsMKK3* haplotypes. *OsMKK3*-Hap1 is the main haplotype in 3K accessions (Figure 4A). A previous study indicated that Guangxi province, southern China is likely the region where cultivated rice was first developed (Huang et al., 2012). To characterize the distribution of *OsMKK3* alleles in wild rice and cultivar accessions, we sequenced *OsMKK3* in 342 Guangxi common wild rice core germplasm accessions, 419 Guangxi core germplasm landrace accessions, and 94 improved varieties (Supplementary Table 1). The results indicated that 278 wild accessions carried *OsMKK3*-Hap1, *OsMKK3*-Hap2, *OsMKK3*-Hap4, and *OsMKK3*-Hap6 at the *OsMKK3* locus. A total of 64 wild accessions showed heterozygous haplotypes or haplotype deficiency at the *OsMKK3* locus. One wild accession with Hap1 and one wild accession with Hap6 were from Laibin (Figure 4B). One wild accession with Hap2 was from Guigang (Figure 4B). Aside from these, all wild accessions had Hap4 of class B. One wild accession had Hap1, Hap2, and Hap6. These findings indicate that there is high diversity at the *OsMKK3* locus. A total of 261 landraces in the putative zone of origin were analyzed. Among the landrace varieties, only one *indica* from Nanning had Hap4 (Figure 4B). A total of 6 *indica* and 3 *japonica* cultivars with Hap1, Hap2, and Hap3 and 5 wild rice accessions were from Guilin in the higher elevation zone (Figure 4B). In higher elevation zones such as Guilin, Liuzhou, Hechi, and Hezhou, *japonica* cultivars accounted for a large proportion of core germplasm accessions (Figure 4B). Hap1, Hap2, and Hap3 were present in improved varieties but not Hap4, Hap5, and Hap6. Almost all *indica* accessions and *japonica* accessions from *Oryza rufipogon* to *O. sativa* had *OsMKK3*-Hap. *indica* cultivars were in the lower elevation zone. *OsMKK3* may have undergone selection from *O. rufipogon* to *O. sativa*; Hap4 was the most common in wild rice compared with Hap1, which was the most common in landrace varieties. Haplotype diversity was decreased in improved varieties.

### *OsMKK3*-Hap1 and Hap2 Are Associated With Longer Grain Length in a GS3/gs3 Background in *Indica* and *Japonica*

**FIGURE 5:**
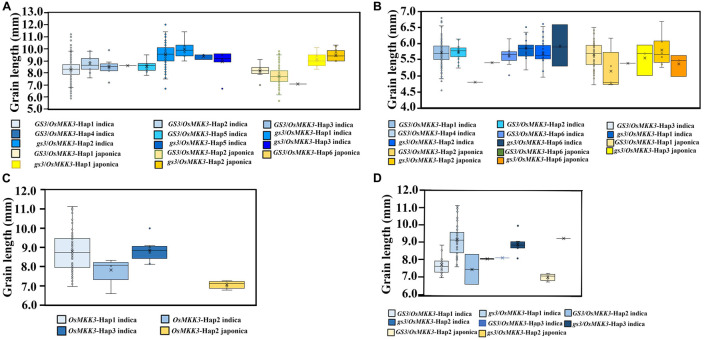
Genetic interactions between *OsMKK3* and other grain length-related genes based on diverse germplasm accessions. Box plots and kernel density plots were generated for different groups. **(A)** Genetic interactions between *OsMKK3* and *GS3*/*gs3* in the rice 3K project. **(B)** Genetic interactions between *OsMKK3* and *GS3*/*gs3* in Guangxi core germplasm landrace accessions. **(C)** Effects of *OsMKK3* in improved varieties. **(D)** Genetic interactions between *OsMKK3* and *GS3*/*gs3* in improved varieties.

To characterize the genetic interaction between *OsMKK3* and *GS3* in controlling grain length, we examined the haplotypes of *OsMKK3* and *GS3* in the rice 3K project, 342 Guangxi common wild rice core germplasm accessions, 419 Guangxi core germplasm landrace accessions, and 94 improved varieties. In the rice 3K project, grain length was longer with *OsMKK3*-Hap2 compared with other *OsMKK3* haplotypes of *indica* in a *GS3* background. Grain length was longer with *OsMKK3*-Hap1 and *OsMKK3*-Hap2 compared with other *OsMKK3* haplotypes of *indica* in a *gs3* background. Grain length was longer with *OsMKK3*-Hap1 compared with other *OsMKK3* haplotypes of *japonica* in a *GS3* background. Grain length was longer with *OsMKK3*-Hap2 compared with other *OsMKK3* haplotypes of *japonica* in a *gs3* background (Figure 5A). *OsMKK3*-Hap1 and *OsMKK3*-Hap2 thus appear to positively affect the grain length in *indica* and *japonica* in a *GS3*/*gs3* background.

**FIGURE 6:**
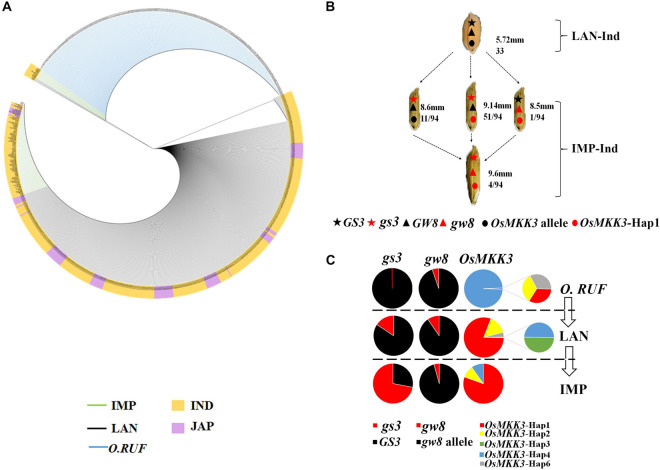
Breeding improvement of *OsMKK3*. **(A)** Phylogenetic analysis of haplotypes of *OsMKK3* in wild rice accessions, Guangxi core germplasm landrace accessions, and improved varieties. The phylogenetic tree of varieties was constructed based on different functional SNPs in *OsMKK3* CDS by MAGE 7.0. The blue line corresponds to wild rice, the black line corresponds to landraces, and the green line corresponds to improved varieties. **(B)** Improvement of the haplotype combination for three grain length-related genes, as in **(A)**. Top numbers indicate average grain length; bottom numbers correspond to grain length-related genes, as in **(A)**. Top numbers indicate average grain length; bottom numbers correspond to the accession number of a certain haplotype combination in the subgroup. **(C)** Spectra of allele frequencies comparing landrace and improved varieties at the causal polymorphisms of *GS3*, *GW8*, and *OsMKK3* during modern breeding in the respective subgroups. LAN landraces, IMP improved varieties.

To investigate the relationship between *OsMKK3* and *GS3* in Guangxi core germplasm landrace accessions, we examined their effects on grain length under different backgrounds. The results indicated that in Guangxi core germplasm landrace accessions, *OsMKK3*-Hap1 and *OsMKK3*-Hap2 positively affected the grain length of *indica*. *OsMKK3*-Hap1 had a stronger effect on the grain length of *japonica* (Figure 5B). In Guangxi core germplasm landrace accessions, the grain length of landraces was longer with *OsMKK3*-Hap1 and *OsMKK3*-Hap2 compared with other *OsMKK3* haplotypes in a *GS3* background of *indica*. Grain length of *OsMKK3*-Hap1 in a *GS3* background significantly differed between *japonica* and *indica.* Furthermore, in the 94 improved varieties, grain length was longer with *OsMKK3*-Hap1 in *indica* (Figures 5C,D). These findings indicate that grain length is regulated by different *OsMKK3* and *GS3* haplotypes. To confirm the observed changes in *OsMKK3* haplotype, we performed a phylogenetic analysis of *OsMKK3* using Guangxi common wild rice core germplasm accessions, Guangxi core germplasm landrace accessions, and improved varieties. Phylogenetic analysis showed that grain length was increased in the improved varieties (Figure 6A). *OsMKK3*-Hap of breeding varieties was concentrated in class A (*OsMKK3*-Hap1, *OsMKK3*-Hap2, and *OsMKK3*-Hap3). Landraces and wild rice accessions were clearly distinguished in *OsMKK3*-Hap.

To determine whether *OsMKK3* was involved in the control of grain size along with other genes, we analyzed the grain length of landraces using different gene haplotypes. Grain length was shorter with *OsMKK3*-Hap (not hap1), *GS3*, and *GW8* than in groups with one more dominant genotype, such as *OsMKK3*-Hap1/*gs3*/*GW8* and *OsMKK3*-Hap1/*GS3*/*gw8*. Grain length increased with *OsMKK3*-Hap1/*gs3*/*gw8* (Figure 6B). These results indicated that the grain length in *indica* rice is improved by the presence of beneficial alleles of *OsMKK3*-Hap1, *gs3*, and *gw8*. *gs3* is a beneficial allele that was only present in 0.31% of wild rice accessions, 15.71% of landrace accessions, and 72.04% of improved varieties. *gw8* was also a beneficial allele that appeared in a few wild rice accessions, landrace accessions, and improved varieties. The *OsMKK3* haplotypes were common in wild rice and included *OsMKK3*-Hap1, *OsMKK3*-Hap2, *OsMKK3*-Hap4, and *OsMKK3*-Hap6. *OsMKK3*-Hap4 was the most common haplotype in wild rice (Figure 6C). The haplotypes of *OsMKK3* were abundant in landraces, and *OsMKK3*-Hap1 was the most common. *OsMKK3*-Hap1 was the most common haplotype in improved varieties, but *OsMKK3*-Hap2 and *OsMKK3*-Hap3 were also observed. Our analysis indicates that *gs3* occurs widely in wild rice, landraces, and improved varieties. The beneficial allele *Gw8* has not been widely used in improved varieties. *OsMKK3*-Hap1 is a beneficial allele that has been used in landraces and improved varieties.

### The Aggregation of Many Beneficial Alleles Improves the Quality of Hybrid Rice

**FIGURE 7:**
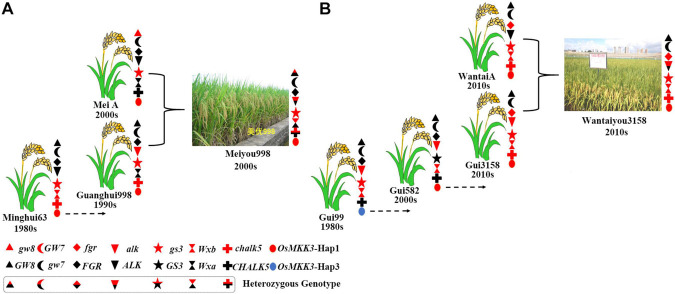
Aggregation of beneficial alleles in hybrid rice in different eras. **(A)** Aggregation of beneficial alleles in the hybrid rice variety Meiyou998 in the 2000s. **(B)** Aggregation of beneficial alleles in the hybrid rice variety Wantaiyou3158 in the 2010s.

To determine the effect of aggregating many beneficial alleles on the quality of hybrid rice, we genotyped a series of hybrid parents of *Alk*, *Wx*, *chalk5*, *fgr*, *gs3*, *gw7*, and *OsMKK3.* Yexiang hybrid rice varieties were included in 16 combinations of hybrid rice (Supplementary Table 4). Yexiang hybrid rice has a ratio of length to width ranging from 2.6 to 4.1, chalkiness degree ranging from 0.1 to 7.5, gel consistency ranging from 53 to 84 mm, and amylose content ranging from 13 to 22.1%. Thus, Yexiang hybrid rice is high quality; it has a planted area of more than 1,088,666 ha (Supplementary Table 4)^6^. We determined the genotypes of some important genes in the 16 combinations of Yexiang hybrid rice. Sterile line Yexiang A had the beneficial alleles *Alk*, *Wx*, *chalk5*, *fgr*, *gs3*, and *OsMKK3*-hap1 (Table 2). The restorer lines R2hao, R456, and R700 had the beneficial alleles *Wx* and *gs3*. R3hao and R803 had the beneficial alleles *Wx*, *chalk5*, *gs3*, and *OsMKK3*-Hap1 (Table 2). Rlisi and Rmingyuesimiao had the beneficial alleles *Alk*, *Wx*, *chalk5*, *gs3*, *gw7*, and *OsMKK3*-Hap1 (Table 2). Rbasi had the greatest number of beneficial alleles (*Alk*, *Wx*, *chalk5*, *fgr*, *gs3*, *gw7*, and *OsMKK3*-Hap1) among the 16 restorer lines (Table 2). The 16 restorer lines contained the three beneficial alleles *wx*, *gs3*, and *OsMKK3*-Hap1/Hap3 (Table 2). The results showed that the quality of hybrid rice can be improved by aggregating more beneficial alleles of important genes. We identified the genotypes *Alk*, *Wx*, *chalk5*, *fgr*, *gs3*, *gw7*, and *OsMKK3* in hybrid parents of the good quality hybrid rice variety Meiyou998 in the 2000s and the super hybrid rice variety Wantaiyou3158 in the 2010s to determine how polymerizing beneficial alleles could be used to breed super rice varieties. Meiyou998 had the following features: ratio of length to width 3.1, chalkiness degree 1.7, gel consistency 67 mm, and amylose content 21%. The sterile line MeiA had beneficial alleles of *gw8*, *gs3*, and *OsMKK3*-Hap1 in the 2000s. Minghui63 had the beneficial alleles *gs3*, *wx*, *chalk5*, and *OsMKK3*-Hap1 in the 1980s. Guanghui998 was bred from Minghui63 in the 1990s with the beneficial alleles *gs3*, *alk, chalk5*, and *OsMKK3*-Hap1 (Figure 7A). Thus, the hybrid rice variety Meiyou998 had the beneficial alleles *gw8*, *alk*, *gs3*, *Wx*, *chalk5*, and *OsMKK3*-Hap1. The results indicated that the quality of the hybrid rice variety Meiyou998 was regulated by *gw8*, *alk*, *gs3*, *Wx*, *chalk5*, and *OsMKK3*-Hap1. In addition, the genotypes of *Alk*, *Wx*, *chalk5*, *fgr*, *gs3*, *gw7*, and *OsMKK3* were identified in the parents of Wantaiyou3158. The super hybrid rice variety Wantaiyou3158 had the following features: ratio of length to width 3.4, chalkiness degree 0.5, gel consistency 78 mm, and amylose content 13.3%. Gui99 had the beneficial alleles *alk*, *gs3*, *Wx*, and *OsMKK3*-Hap3 in the 1980s. In the 2000s, Gui582 was bred from Gui99 with *alk*, *wxb*, and *OsMKK3*-Hap1. In the 2010s, Gui3158 was bred from Gui582 with the beneficial alleles *fgr*, *wx*, *alk*, *gs3*, *chalk*, and *OsMKK3*-Hap1. The sterile line WantaiA had the beneficial alleles *fgr*, *gs3*, *wx*, *chalk*, and *OsMKK3*-Hap1 in the 2010s (Figure 7B). Thus, the hybrid rice variety Wantaiyou3158 had the beneficial alleles *fgr*, *wx*, *alk*, *gs3*, *chalk*, and *OsMKK3*-Hap1 in the 2010s. These results shed light on how the quality of hybrid rice has improved from the 1980s to now. The quality of hybrid rice was improved by a greater number of beneficial alleles when *OsMKK3*-Hap1 was aggregated in the sterile line and restorer line. The presence of beneficial alleles of important genes in homozygous form has greatly improved the quality of hybrid rice. *OsMKK3*-Hap1 had a particularly pronounced positive effect in improving the quality of hybrid rice.

**TABLE 2:** Genotype of restorer lines.

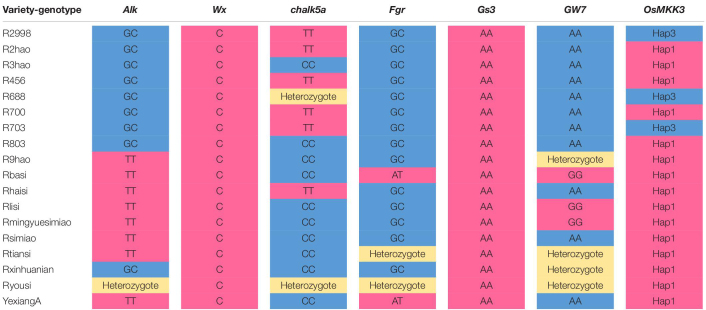

*Alk-TT, Wx-c, chalk5a-cc, Fgr-AT, GS3-AA, and GW7-GG are beneficial alleles. Peach is beneficial alleles, blue is not beneficial alleles, yellow is heterozygote.*

## Discussion

Exploring the genes involved in the production and quality of rice and employing them under an appropriate genetic background remains a major challenge. Grain size is one of the key factors associated with grain weight, which affects rice yield. Several genes for grain size such as *GS3*, *GL3.1*, *GW6a*, *OsLG3*, *OsLG3b*, *gw8*, *GS5*, *SMG1*, and *OsSPL13* have been cloned from natural rice varieties (Fan et al., 2006; Yan et al., 2011; Wang et al., 2012; Li et al., 2014; Si et al., 2016; Xia et al., 2018; Yu et al., 2018). The genes involved in regulating the quality of rice such as *chalk5* (Li et al., 2014), *fgr* (Bradbury et al., 2005), and *wx* (Zhou et al., 2016) have been used in breeding by molecular design. In this study, we showed that *OsMKK3* is a positive regulator of grain size and chalkiness. It controls the grain length by enhancing cell proliferation in spikelet hulls. Genes with natural variations that regulate important agronomic traits have been used by breeders to improve crop yield and quality.

### Identifying Pleiotropy Is Essential for Gene Mining and Utilization

There are two types of genes with genic pleiotropy: one is a gene with a single function but involved in multiple biological processes, such as *fgr* (Bradbury et al., 2005), *wx* (Zhou et al., 2016), and *chalk5* (Li et al., 2014). Another is a gene with multiple functions that contributes to different traits (Chen et al., 2010), such as *GS3* (Takano-Kai et al., 2009), *Hd1* (Leng et al., 2020), and *GS5* (Li et al., 2011). Additional difficulties associated with utilizing pleiotropic genes stem from the effect of adverse correlations between favorable traits and disadvantageous traits (Li et al., 2018). Identifying the function of new genes and obtaining information on new genes is essential for developing optimal utilization strategies. A loss-of-function mutation of *GW8*/*OsSPL16* in Basmati rice is associated with the formation of a slender grain and better appearance quality (Wang et al., 2012). *GL7*/*GW7* plays a role in grain size and chalkiness; the *OsSPL16*-*GW7* module regulates cell division and rice endosperm (Wang et al., 2015). *qSW5*/*GW5*/*GSE5* results in grain width diversity in rice and salt stress resistance through an association with the calmodulin protein *OsCaM1−1* (Tian et al., 2019). *Ghd8*/*DTH8* likely plays an important role in the signal network of photoperiodic flowering as a novel suppressor as well as in the regulation of plant height and yield potential (Wei et al., 2010). *TGW6* encodes a novel protein with indole-3-acetic acid (IAA)-glucose hydrolase activity; control of the IAA supply limits the number of cells and grain length (Ishimaru et al., 2013). *FLO2* plays an important role in regulating rice grain size and starch quality by affecting the accumulation of storage substances. Gong et al. (2017) used large populations of hybrid rice for genetic dissections of grain quality traits and recovered only very weak correlations between the grain length-to-width ratio and degree of chalkiness (Chen et al., 2020). Exploration of the genes involved in the regulation of rice yield and quality provides valuable information that could be used in future research. Identifying the function of genes involved in production and quality could also be used to improve the balance between rice yield and quality. In this study, we showed that *OsMKK3* encodes a MAP Kinase Kinase 3 that regulates the grain length, chalky rice rate, and chalkiness degree. Overexpression of *OsMKK3* in Nip plants increased the quality, grain length, and percentage of grain with chalkiness. The relationship between grain size and chalkiness is complex, which suggests that the genetic architectures of grain size and chalkiness differ. The discovery of new genes provided a genetic resource for rice yield and quality breeding. Based on the observed pleiotropy of genes, the relationships between genes should be an important future direction of breeding efforts.

### Breeding Strategies Need to Be Tailored to Specific Breeding Objectives and Based on Mined Genes

Several hundred QTLs of rice yield and quality have been identified. Mining genes and identifying the haplotypes associated with rice yield and quality provide a foundation for molecular design breeding. Leng et al. (2020) detected 19 haplotypes in *Hd1* and analyzed five major haplotypes in 123 major rice varieties, as well as the relationship between *Hd1* alleles and yield-related traits. Backcrossing of the most preponderant allele *Hap16* into the *japonica* variety Chunjiang06 improved yield without decreasing grain quality. Zhang et al. (2020) found that the null alleles were enriched in modern *indica*, and they introgressed the null *sd1* allele into the elite *japonica* variety Daohuaxiang to improve the semi-dwarf line. Long-grain alleles of *OsLG3b* still have much breeding potential for increasing the grain length in *indica* (Yu et al., 2018). The long-grain allele of *OsLG3* might have much potential value for improving the grain length in *japonica* breeding (Yu et al., 2017). *GSE5* regulates grain size by affecting cell proliferation in spikelet hulls, which indicates that natural variation in the promoter region of *GSE5* contributes to grain size diversity in rice (Duan et al., 2017). Molecular design breeding has been used to enhance the effects of haplotypes and rice breeding based on the mining of genes.

A previous study showed that rational design is a powerful strategy for addressing the challenges of future crop breeding, particularly the pyramiding of multiple complex traits (Zeng et al., 2017). Molecular design breeding models often differ in the balance that they achieve between yield and quality. Wang et al. (2015) developed new high-yielding *indica* hybrid rice varieties with improved yield and grain quality. Wang et al. (2012) converted a line with short and wide grains into one with slender grains by pyramiding the non-functional alleles *gs3* and *gw8* in line HJX74, which substantially improved grain quality. Zeng et al. (2017) pyramided genes that significantly contribute to grain quality and yield from *Nip* (NPB), Teqing, and LYP9 and developed new elite varieties. Wu et al. (2018) developed near-isogenic lines with a background of 9311, and 9311-*OsMADS1*^*lgy*3^ plants carrying haplotype 2 produced much slender and heavier grains than both 9311-*OsMADS1*^*NIP*^ and 9311-*OsMADS1*^9311^ plants. Li et al. (2020) pyramided the favorable haplotypes of five cloned rice grain quality genes, which resulted in a line with very low amylose content and high yield. Multiple patterns of gene aggregation and interaction require different practical strategies for molecular design breeding. The effect of gene aggregation in different backgrounds is an important research direction.

The increasing requirements of consumers underscore the need to develop both high-yield and superior quality rice (Zhang, 2007; Suwannaporn and Linnemann, 2008). Different breeding strategies have been developed for different breeding objectives. Breeding strategies should focus on yield-associated genes to breed high-yield rice. *Gn1a* and *SCM2* control grain number (Ashikari et al., 2005; Ookawa et al., 2010), *TAC1* and *sd1* determine plant architecture (Yu et al., 2007), *Hd1* increases the biomass of most *indica* varieties under short-day conditions (Yano et al., 2000), and *Ghd7* and *Ghd8* regulate grain yield, heading date, and plant height (Xue et al., 2008; Yan et al., 2011). Backbone parents with excellent properties should be used for breeding.

Breeding high-quality rice requires selecting quality genes, such as starch synthesis-related genes (SSRGs) (Tian et al., 2009), *GS3*, and *qSW5*, which control grain shape and grain weight (Fan et al., 2006; Shomura et al., 2008). *Fgr* is associated with the presence of 2-acetyl-1-pyrroline (Bradbury et al., 2005). *Chalk5* encodes a vacuolar H+-translocating pyrophosphatase that affects grain chalkiness in rice (Li et al., 2014). Breeding high quality and environmentally adaptable rice requires balancing resistance genes and quality genes. In this study, we showed that *OsMKK3* regulates grain size and chalkiness, but this gene still has much breeding potential for improving yield and quality when it is polymerized with other benefited alleles. The diversity of breeding strategies is likely to increase with additional research, which will potentially increase the difficulty of mining genes and characterizing their interactions and collocation patterns.

## Conclusion

We cloned and identified *OsMKK3*, which encodes a MAP kinase kinase that controls grain size and chalkiness. *OsMKK3* regulates many genes including *OsSPL16, GS5, GIF1*, and *Wx* at the early spike development stage. *OsMKK3* has undergone a selective sweep during *indica* and temperate *japonica* domestication. Hap4 is the main haplotype in wild rice, and Hap1 is the main haplotype in cultivated rice. In hybrid rice with good yield and quality, *OsMKK3-*Hap1 was polymerized with beneficial alleles. Our findings confirmed that *OsMKK3* enhances crop yield and quality.

## Data Availability Statement

The original contributions presented in the study are publicly available. This data can be found here: National Center for Biotechnology Information (NCBI) BioProject database under accession number PRJNA769799.

## Author Contributions

GDe, DL, GDa, and LG designed and supervised the research. LC and YZ performed the experiments. HG, JL, HW, DQ, and CL analyzed the data. WZ and XY bred the rice variety. YL provided wild rice. YP wrote the manuscript. All authors read and approved the final manuscript.

## Conflict of Interest

XY was employed by Guangxi Lvhai Seed Co., Ltd. The remaining authors declare that the research was conducted in the absence of any commercial or financial relationships that could be construed as a potential conflict of interest.

## Publisher’s Note

All claims expressed in this article are solely those of the authors and do not necessarily represent those of their affiliated organizations, or those of the publisher, the editors and the reviewers. Any product that may be evaluated in this article, or claim that may be made by its manufacturer, is not guaranteed or endorsed by the publisher.

## Footnotes

1
http://www.rmbreeding.cn/Index/s

2
http://www.snpway.com/snpdecoder/

3
http://www.majorbio.com/

4
http://viewer.shigen.info/oryzagenome/

5
http://rice.plantbiology.msu.edu/index.shtml

6
https://www.ricedata.cn/variety/

## Abbreviations

Nip: Nipponbare
QTLs: many quantitative trait loci
PPKL: protein phosphatase kelch
gcHap: gene-coding sequence-haplotype
CaMV35S: tobacco cauliflower mosaic virus
N-OE: Overexpression constructs containing the *OsMKK3* CDS from *Nip*
YXB: Yexiang maintainer line
NT: no transgene line
qRT-PCR: real-time quantitative PCR
Cr: transgenic plants by CRISPR-Cas9 system
DEGs: differentially expressed genes
Hap: haplotypes
MKK3: MAP kinase kinase 3.

## References

[B1] Alexandrov N. Tai S. Wang W. Mansueto L. Palis K. Fuentes R. R. (2015). SNP-Seek database of SNPs derived from 3000 rice genomes. *Nucleic Acids Res.* 43 D1023–D1027. 10.1093/nar/gku1039 25429973 PMC4383887

[B2] Ashikari M. Sakakibara H. Lin S. Yamamoto T. Takashi T. Nishimura A. (2005). Cytokinin oxidase regulates rice grain production. *Science* 309 741–745. 15976269 10.1126/science.1113373

[B3] Bradbury L. M. T. Fitzgerald T. L. Henry R. J. Jin Q. Waters D. L. E. (2005). The gene for fragrance in rice. *Plant Biotechnol. J.* 3 363–370. 17129318 10.1111/j.1467-7652.2005.00131.x

[B4] Chen L. Wang Q. Tang M. Zhang X. Pan Y. Yang X. (2021). QTL mapping and identification of candidate genes for heat tolerance at the flowering stage in rice. *Front. Genet.* 11:621871. 10.3389/fgene.2020.621871 33552136 PMC7862774

[B5] Chen W. Cheng Z. Liu L. Wang M. You X. Wang J. (2010). Molecular basis of trait correlations. *Trends Plant Sci.* 15 454–461. 20542719 10.1016/j.tplants.2010.05.004

[B6] Chen Y. Zhu A. Xue P. Wen X. Cao Y. Wang B. (2020). Effects of *GS3* and *GL3.1* for grain size editing by CRISPR/Cas9 in rice. *Rice Sci.* 27 405–413.

[B7] Danecek P. Auton A. Abecasis G. Albers C. A. Banks E. DePristo M. A. (2011). The variant call format and VCFtools. *Bioinform.* 27 2156–2158. 10.1093/bioinformatics/btr330 21653522 PMC3137218

[B8] Duan P. Rao Y. Zeng D. Yang Y. Xu R. Zhang B. (2014). *SMALL GRAIN 1*, which encodes a mitogen-activated protein kinase kinase 4, influences grain size in rice. *Plant J.* 77 547–557. 10.1111/tpj.12405 24320692

[B9] Duan P. Xu J. Zeng D. Zhang B. Geng M. Zhang G. (2017). Natural variation in the promoter of *GSE5* contributes to grain size diversity in rice. *Mol. Plant* 10 685–694. 10.1016/j.molp.2017.03.009 28366824

[B10] Fan C. Xing Y. Mao H. Lu T. Han B. Xu C. (2006). *GS3*, a major QTL for grain length and weight and minor QTL for grain width and thickness in rice, encodes a putative transmembrane protein. *Theor. Appl. Genet.* 112 1164–1171. 10.1007/s00122-006-0218-1 16453132

[B11] Gong J. Miao J. Zhao Y. Zhao Q. Feng Q. Zhan Q. (2017). Dissecting the genetic basis of grain shape and chalkiness traits in hybrid rice using multiple collaborative populations. *Mol. Plant* 10 1353–1356. 10.1016/j.molp.2017.07.014 28803900

[B12] Heim M. Meyer U. A. (1990). Genotyping of poor metabolisers of debrisoquine by allele-specific PCR amplification. *Lancet* 336 529–532. 10.1016/0140-6736(90)92086-w 1975039

[B13] Huang X. Kurata N. Wei X. Wang Z. Wang A. Zhao Q. (2012). A map of rice genome variation reveals the origin of cultivated rice. *Nature* 490 497–501. 10.1038/nature11532 23034647 PMC7518720

[B14] Ishimaru K. Hirotsu N. Madoka Y. Murakami N. Hara N. Onodera H. (2013). Loss of function of the IAA-glucose hydrolase gene *TGW6* enhances rice grain weight and increases yield. *Nat. Genet.* 45 707–711. 10.1038/ng.2612 23583977

[B15] Kumar S. Stecher G. Tamura K. (2016). MEGA7: molecular evolutionary genetics analysis Version 7.0 for bigger datasets. *Mol. Biol. Evol.* 33 1870–1874. 10.1093/molbev/msw054 27004904 PMC8210823

[B16] Leng Y. Gao Y. Chen L. Yang Y. Huang L. Dai L. (2020). Using *Heading date 1* preponderant alleles from indica cultivars to breed high-yield, high-quality japonica rice varieties for cultivation in south China. *Plant Biotechnol. J.* 18 119–128. 10.1111/pbi.13177 31141272 PMC6920332

[B17] Letunic I. Bork P. (2019). Interactive Tree Of Life (iTOL) v4: recent updates and new developments. *Nucleic Acids Res.* 47 W256–W259. 10.1093/nar/gkz239 30931475 PMC6602468

[B18] Li F. Xie J. Zhu X. Wang X. Zhao Y. Ma X. (2018). Genetic basis underlying correlations among growth duration and yield traits revealed by GWAS in rice (*Oryza sativa* L.). *Front. Plant Sci.* 9:650. 10.3389/fpls.2018.00650 29872443 PMC5972282

[B19] Li L. Zheng X. Zhang X. Xu K. Song S. Su J. (2020). Combination of favorable gene haplotypes plays an important role in rice hybrid grain quality traits based on genome-wide association analysis. *bioRxiv* 10.1101/2020.06.04.134023

[B20] Li Y. Fan C. Xing Y. Jiang Y. Luo L. Sun L. (2011). Natural variation in *GS5* plays an important role in regulating grain size and yield in rice. *Nat. Genet.* 43 1266–1269. 10.1038/ng.977 22019783

[B21] Li Y. Fan C. Xing Y. Yun P. Luo L. Yan B. (2014). *Chalk5* encodes a vacuolar H(+)–translocating pyrophosphatase influencing grain chalkiness in rice. *Nat. Genet.* 46 398–404. 10.1038/ng.2923 24633159

[B22] Librado P. Rozas J. (2009). DnaSP v5: a software for comprehensive analysis of DNA polymorphism data. *Bioinform.* 25 1451–1452. 10.1093/bioinformatics/btp187 19346325

[B23] Liu Q. Han R. Wu K. Zhang J. Ye Y. Wang S. (2018). G-protein βγ subunits determine grain size through interaction with MADS-domain transcription factors in rice. *Nat. Commun.* 9:852. 10.1038/s41467-018-03047-9 29487282 PMC5829230

[B24] Mao H. Sun S. Yao J. Wang C. Yu S. Xu C. (2010). Linking differential domain functions of the *GS3* protein to natural variation of grain size in rice. *Proc. Natl. Acad. Sci. U.S.A.* 107 19579–19584. 10.1073/pnas.1014419107 20974950 PMC2984220

[B25] Mao Y. Zhang H. Xu N. Zhang B. Gou F. Zhu J. K. (2013). Application of the CRISPR-Cas system for efficient genome engineering in plants. *Mol. Plant* 6 2008–2011. 10.1093/mp/sst121 23963532 PMC3916745

[B26] Newton C. R. Graham A. Heptinstall L. E. Powell S. J. Summers C. Kalsheker N. (1989). Analysis of any point mutation in DNA. The amplification refractory mutation system (ARMS). *Nucleic Acids Res.* 17 2503–2516. 2785681 10.1093/nar/17.7.2503 PMC317639

[B27] Ookawa T. Hobo T. Yano M. Murata K. Ando T. Miura H. (2010). New approach for rice improvement using a pleiotropic QTL gene for lodging resistance and yield. *Nat. Commun.* 1:132. 10.1038/ncomms1132 21119645 PMC3065348

[B28] Pan Y. Xu Z. Liang Y. (2018). Genetic structure and core collection of common wild rice (*Oryza rufipogon* Griff.) in Guangxi. *J. Plant Genet.* 19 498–509.

[B29] Pan Y. H. Gao L. J. Liang Y. T. Zhao Y. Liang H. F. Chen W. W. (2021). *OrMKK3* influences morphology and grain size in rice. *J. Plant Biol.* Jan 4 1–14. 10.1007/s12374-020-09290-2 33424241 PMC7780602

[B30] Qi P. Lin Y. S. Son X. J. Shen J. B. Huang W. Shan J. X. (2012). The novel quantitative trait locus *GL3.1* controls rice grain size and yield by regulating Cyclin-T1;3. *Cell Res.* 22 1666–1680. 10.1038/cr.2012.151 23147796 PMC3515756

[B31] Shomura A. Izawa T. Ebana K. Ebitani T. Kanegae H. Konishi S. (2008). Deletion in a gene associated with grain size increased yields during rice domestication. *Nat. Genet.* 40 1023–1028. 10.1038/ng.169 18604208

[B32] Si L. Chen J. Huang X. Gong H. Luo J. Hou Q. (2016). *OsSPL13* controls grain size in cultivated rice. *Nat. Genet.* 48 447–456. 10.1038/ng.3518 26950093

[B33] Song X. J. Kuroha T. Ayano M. Furuta T. Nagai K. Komeda N. (2015). Rare allele of a previously unidentified histone H4 acetyltransferase enhances grain weight, yield, and plant biomass in rice. *Proc. Natl. Acad. Sci. U.S.A.* 112 76–81. 10.1073/pnas.1421127112 25535376 PMC4291654

[B34] Suwannaporn P. Linnemann A. R. (2008). Rice-eating quality among consumers in different rice grain preference countries. *J. Sens. Stud.* 23 1–13. 10.1111/j.1745-459x.2007.00129.x

[B35] Takano-Kai N. Jiang H. Kubo T. Sweeney M. Matsumoto T. (2009). Evolutionary history of *GS3*, a gene conferring grain length in rice. *Genet.* 182 1323–1334. 10.1534/genetics.109.103002 19506305 PMC2728869

[B36] Tian P. Liu J. Mou C. Shi C. Zhang H. Zhao Z. (2019). *GW5*-Like, a homolog of *GW5*, negatively regulates grain width, weight and salt resistance in rice. *J. Integr. Plant Biol.* 61 1171–1185. 10.1111/jipb.12745 30450718

[B37] Tian Z. Qian Q. Liu Q. Yan M. Liu X. Yan C. (2009). Allelic diversities in rice starch biosynthesis lead to a diverse array of rice eating and cooking qualities. *Proc. Natl. Acad. Sci. U.S.A.* 106 21760–21765. 10.1073/pnas.0912396106 20018713 PMC2793318

[B38] Wang S. Li S. Liu Q. Wu K. Zhang J. Wang S. (2015). The *OsSPL16-GW7* regulatory module determines grain shape and simultaneously improves rice yield and grain quality. *Nat. Genet.* 47 949–954. 10.1038/ng.3352 26147620

[B39] Wang S. Wu K. Yuan Q. Liu X. Liu Z. Lin X. (2012). Control of grain size, shape and quality by *OsSPL16* in rice. *Nat. Genet.* 44 950–954. 10.1038/ng.2327 22729225

[B40] Wang W. Mauleon R. Hu Z. Chebotarov D. Tai S. Wu Z. L. (2018). Genomic variation in 3,010 diverse accessions of Asian cultivated rice. *Nature* 557 43–49. 10.1038/s41586-018-0063-9 29695866 PMC6784863

[B41] Wei X. Xu J. Guo H. Jiang L. Chen S. Yu C. (2010). *DTH8* suppresses flowering in rice, influencing plant height and yield potential simultaneously. *Plant Physiol.* 153 1747–1758. 10.1104/pp.110.156943 20566706 PMC2923886

[B42] Wu K. Xu X. Zhong N. Huang H. Yu J. Ye Y. (2018). The rational design of multiple molecular module-based assemblies for simultaneously improving rice yield and grain quality. *J.Genet. Genomics* 45 337–341. 10.1016/j.jgg.2018.03.007 29929851

[B43] Xia D. Zhou H. Liu R. Dan W. Li P. Wu B. (2018). *GL3.3*, a novel QTL encoding a GSK3/SHAGGY-like Kinase, epistatically interacts with *GS3* to produce extra-long grains in rice. *Mol. Plant* 11 754–756. 10.1016/j.molp.2018.03.006 29567448

[B44] Xue W. Xing Y. Weng X. Zhao Y. Tang W. Wang L. (2008). Natural variation in *Ghd7* is an important regulator of heading date and yield potential in rice. *Nat. Genet.* 40 761–767. 10.1038/ng.143 18454147

[B45] Yan W. H. Wang P. Chen H. X. Zhou H. J. Li Q. P. Wang C. R. (2011). A major QTL, *Ghd8*, plays pleiotropic roles in regulating grain productivity, plant height, and heading date in rice. *Mol. Plant* 4 319–330. 10.1093/mp/ssq070 21148627

[B46] Yang X. Xia X. Zeng Y. Nong B. Zhang Z. Wu Y. (2018). Identification of candidate genes for gelatinization temperature, gel consistency and pericarp color by GWAS in rice based on SLAF-sequencing. *PLo*S *One* 13:e0196690. 10.1371/journal.pone.0196690 29746484 PMC5944943

[B47] Yano M. Katayose Y. Ashikari M. Yamanouchi U. Monna L. Fuse T. (2000). Hd1, a major photoperiod sensitivity quantitative trait locus in rice, is closely related to the *Arabidopsis* flowering time gene *CONSTANS*. *Plant Cell* 12 2473–2484. 10.1105/tpc.12.12.2473 11148291 PMC102231

[B48] Yu B. Lin Z. Li H. Li X. Li J. Wang Y. (2007). *TAC1*, a major quantitative trait locus controlling tiller angle in rice. *Plant J.* 52 891–898. 10.1111/j.1365-313X.2007.03284.x 17908158

[B49] Yu J. Miao J. Zhang Z. Xiong H. Zhu X. Sun X. (2018). Alternative splicing of *OsLG3b* controls grain length and yield in japonica rice. *Plant Biotechnol. J.* 16 1667–1678. 10.1111/pbi.12903 29479793 PMC6097128

[B50] Yu J. Xiong H. Zhu X. Zhang H. Li H. Miao J. (2017). *OsLG3* contributing to rice grain length and yield was mined by Ho-LAMap. *BMC Biol.* 15:28. 10.1186/s12915-017-0365-7 28385155 PMC5383996

[B51] Zeng D. Tian Z. Rao Y. Dong G. Yang Y. Huang L. (2017). Rational design of high-yield and superior-quality rice. *Nat. Plants* 3:17031. 10.1038/nplants.2017.31 28319055

[B52] Zhang F. Wang C. Li M. Cui Y. Shi Y. Wu Z. (2021). The landscape of gene–CDS–haplotype diversity in rice: properties, population organization, footprints of domestication and breeding, and implications for genetic improvement. *Mol. Plant* 14 11–18. 10.1016/j.molp.2021.02.003 33578043

[B53] Zhang J. Wang J. Wang J. Zhao Z. Guo X. Lei C. (2019). Small Grain and *Dwarf 2*, encoding an HD-Zip II family transcription factor, regulates plant development by modulating gibberellin biosynthesis in rice. *Plant Sci.* 288 110208. 10.1016/j.plantsci.2019.110208 31521223

[B54] Zhang L. Bian Z. Ma B. Li X. Zou Y. Xie D. (2020). Exploration and selection of elite Sd1 alleles for rice design breeding. *Mol. Breed.* 40:79.

[B55] Zhang Q. (2007). Strategies for developing Green super rice. *Proc. Natl. Acad. Sci. U.S.A.* 104 16402–16409. 10.1073/pnas.0708013104 17923667 PMC2034246

[B56] Zhang X. Wang J. Huang J. Lan H. Wang C. Yin C. (2012). Rare allele of *OsPPKL1* associated with grain length causes extra-large grain and a significant yield increase in rice. *Proc. Natl. Acad. Sci. U.S.A.* 109 21534–21539. 10.1073/pnas.1219776110 23236132 PMC3535600

[B57] Zhou H. Wang L. Liu G. Meng X. Jing Y. Shu X. (2016). Critical roles of soluble starch synthase SSIIIa and granule-bound starch synthase Waxy in synthesizing resistant starch in rice. *Proc. Natl. Acad. Sci. U.S.A.* 113 12844–12849. 10.1073/pnas.1615104113 27791174 PMC5111662

[B58] Zhou W. Chen W. Dai G. Liang H. Zhou M. Chen R. (2019). Wantaiyou 3158, a new super hybrid rice combination for both early and late season. *Hybrid Rice* 34, 80–81. 10.16267/j.cnki.1005-3956.20190226.049

